# Supplementary material for: Mapping electric fields and observation of ferroelectric domain switching in hafnia-zirconia devices by electron holography
Source: Nat Commun. 2025 Dec 18;16:11233. doi: 10.1038/s41467-025-66807-4 (PMC12715209; doi:10.1038/s41467-025-66807-4)
Supplement: Supplementary file 1 — Supplementary information [file 41467_2025_66807_MOESM1_ESM.pdf]

Supplementary information for

Mapping electric fields and observation of ferroelectric domain switching in hafnia-zirconia devices by electron holography

Leifeng Zhang[1], Christophe Gatel*[1,2], Muhammad Hamid Raza[3], Kilian Gruel[1], Catherine Dubourdieu*[3,4], Martin Hÿtch[1]

[1] CEMES-CNRS and Université de Toulouse, 29 rue Jeanne Marvig, 31055 Toulouse, France

[2] Université Paul Sabatier, 31062 Toulouse, France

[3] Helmholtz-Zentrum Berlin für Materialien und Energie, Hahn-Meitner-Platz 1, 14109 Berlin, Germany

[4] Freie Universität Berlin, Physical and Theoretical Chemistry, Arnimallee 22, 14195 Berlin, Germany

*Corresponding author:

christophe.gatel@cemes.fr

catherine.dubourdieu@helmholtz-berlin.de

Figure S1 presents the P-V curve from positive-up negative-down (PUND) measurement, which shows the hysteresis characteristic. Prior to electrical tests, the fabricated FTJs were woken up through 5000 cycles of a triangular waveform at 1 kHz with a 4.5 V amplitude. Electron holograms were then recorded at different applied biases following the remnant P-V curve.

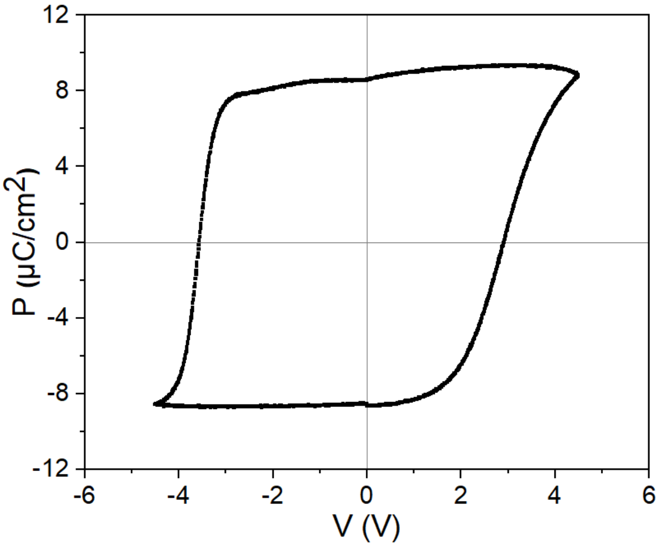

Fig. S1: P-V curve from PUND measurement of the TiN/9 nm HZO/2.5 nm Al_2O_3/W bilayer FTJ device

Dedicated sample preparation is key to *operando* electrical biasing TEM experiments. Specimens for *operando* electron holography experiments were prepared by focused ion beam (FIB, Ga^+ source, Helios 600i from FEI), by which a lamella device is constructed on a commercialized Hummingbird chip compatible with biasing holder (1600 series, Hummingbird scientific). The Hummingbird holder, chip as well as the FIB-prepared specimen devices are shown in Fig. S2. Figure S2a illustrates the Hummingbird sample holder with the inserted Hummingbird chip. The architecture of the chip is shown in Fig. S2b. It has 9 conductive, separate Au tracks (as labelled from No. 1 to 9), perfectly compatible with the 9 separate Au contacts in the Hummingbird biasing holder. The insert of Fig. S2b shows its working window, and the FIB-prepared lamellae could be placed between Au paws 3 and 7. The designed device specimen architecture and the FIB-fabricated sample device are displayed in Fig. S2c and Fig. S2d, respectively. During sample preparation, a large lamella embedding the structure of interest has been milled and lift-out from the bulk device and welded onto the Au tracks of the chip. After thinning of the lamellae, FIB undercuts were done on its left and right sides to eliminate short-circuits from top and bottom electrodes. The sample preparation was finished with a cleaning at 1 kV, 28 pA. For TEM/STEM characterizations, standard lamellas were prepared by FIB typical protocols.

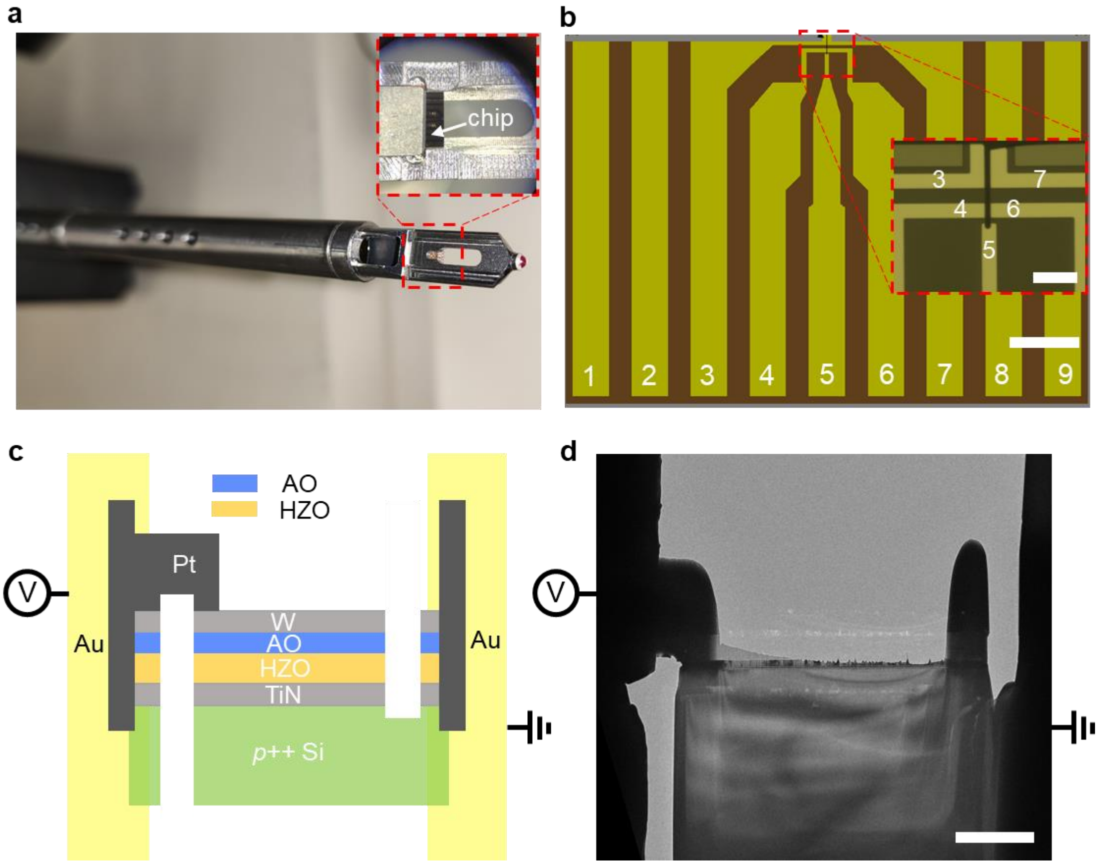

Fig. S2: Biasing specimen holder, chip and FIB-prepared device. a Hummingbird specimen holder and the inset showing a chip being inserted into the holder. **b** the Hummingbird chip with its working space for FIB lift-out (in the inset). **c** Schematic illustration of the device architecture. **d** FIB-prepared sample device. At the back of the Hummingbird biasing holder are connected cables allowing electrical signal input from external source generator. Scale bars in **b** and its inset are 400 μm and 50 μm, respectively. Scale bar is 1 μm in **d**.

Figures S3-S5 show the bright field (BF)-STEM, dark field (DF)-STEM, and high resolution (HR)-TEM images. In Fig. S4, the BF-STEM, DF-STEM and HRTEM images were captured from the same region. From these correlative TEM and STEM characterizations, this interfacial layer (IL), is structurally invisible at the interface between ferroelectric HZO and TiN electrode.

Besides, these images confirm the polycrystalline nature of the deposited HZO film, and the orthorhombic HZO polar phase (space group: $Pca2_1$) is clearly identified in Figs. S4 and S5.

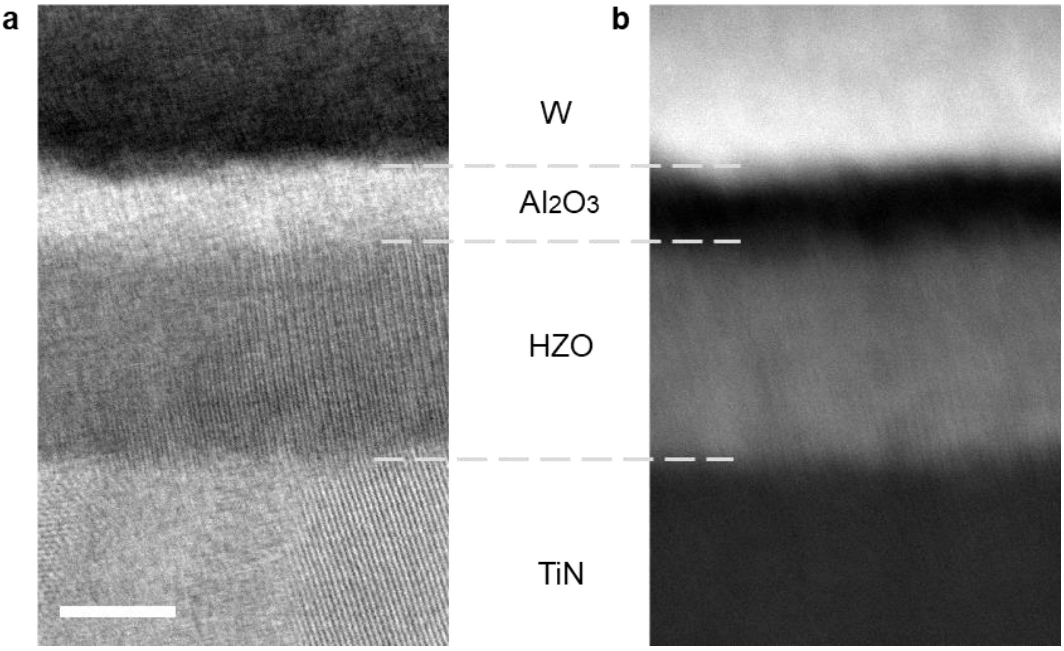

Fig. S3: STEM images showing the microstructure of the bilayer FTJ device. a BF-STEM image. **b** HAADF-STEM image. The interfacial layer (IL) at the TiN-HZO interface is structurally indistinguishable. Scale bar is 5 nm.

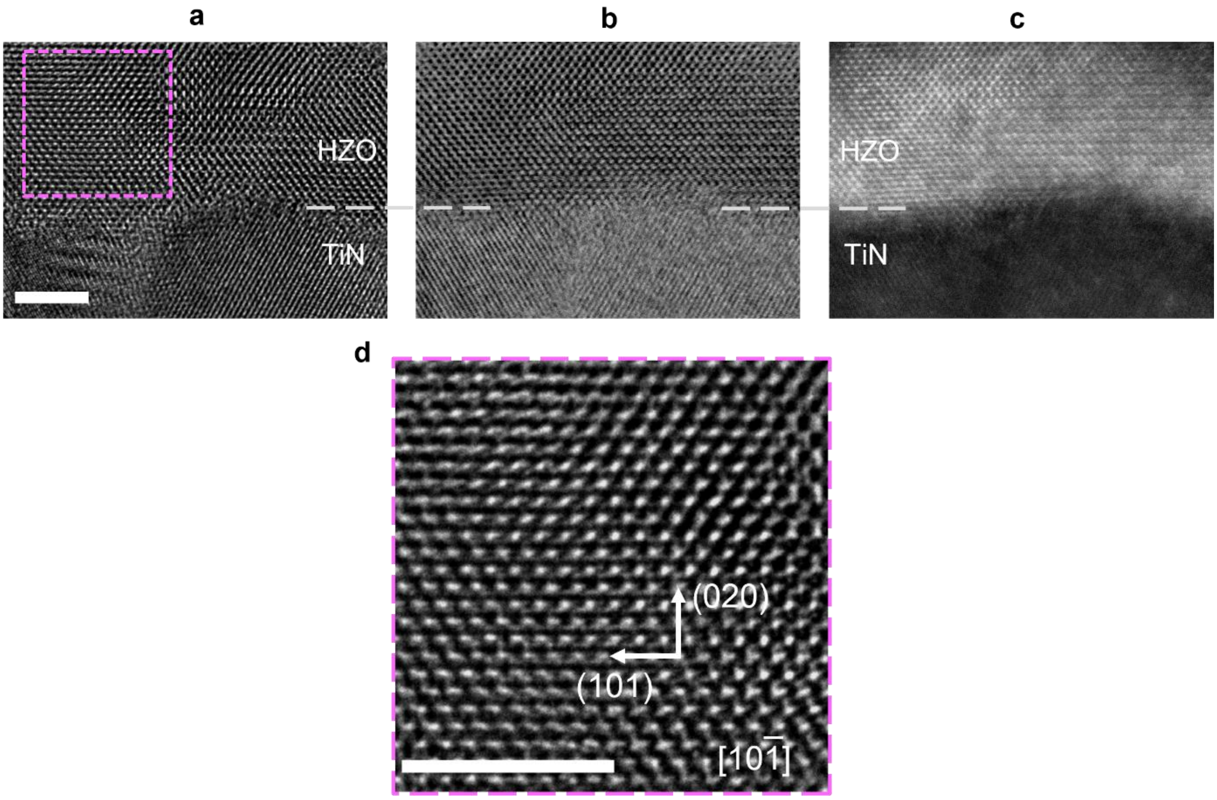

Fig. S4: HR-TEM, BF-STEM and HAADF-STEM images showing the microstructure of the bilayer FTJ device. **a** HR-TEM image. **b** BF-STEM image. **c** DF-STEM image on the same area than **b**. **d** Local magnification image of the marked region with pink dashed rectangular in **a** showing the orthorhombic HZO polar phase (space group: $Pca2_1$). The interfacial layer (IL) at the TiN-HZO interface is structurally invisible. Scale bars are 3 nm in **a** and **d**.

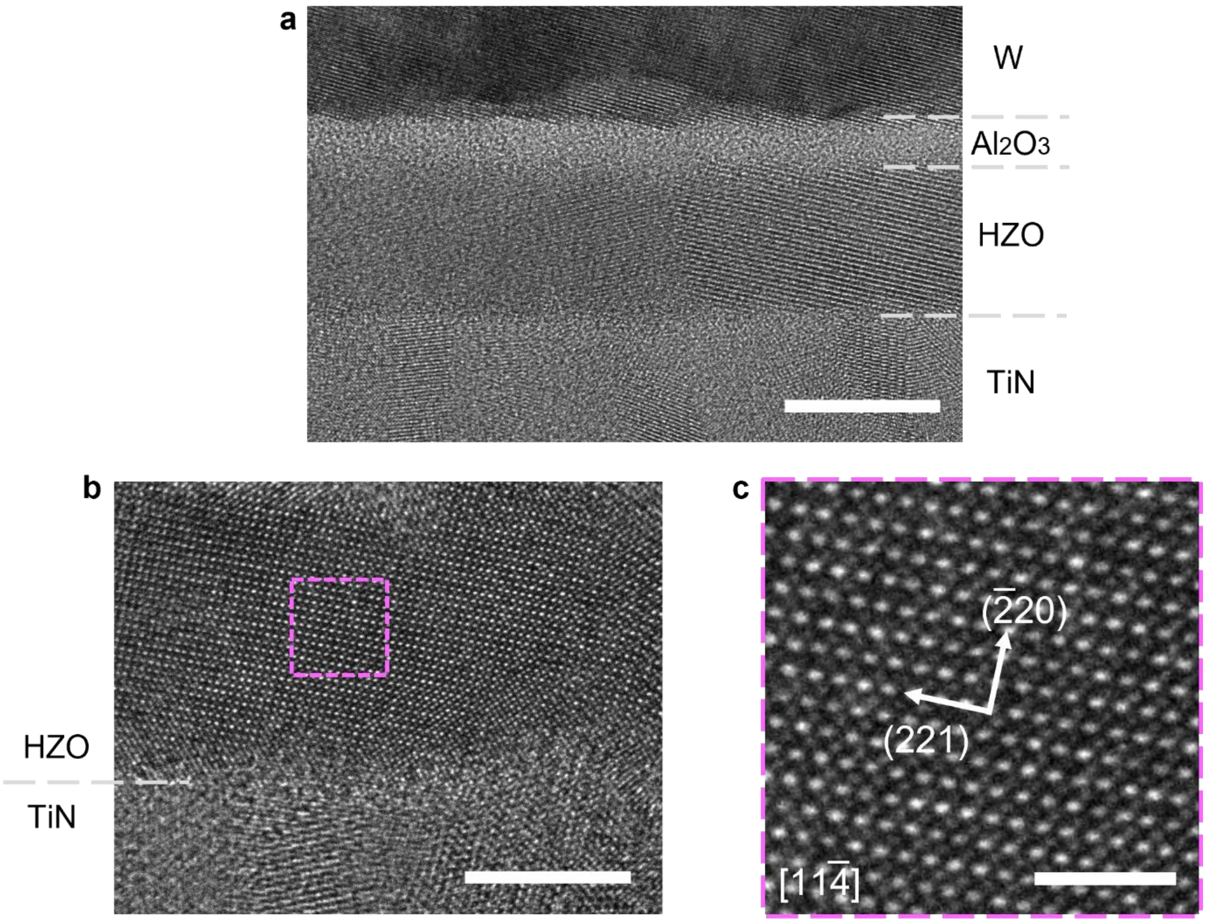

Fig. S5: HRTEM cross-section images of the bilayer FTJ stack. a Cross-section image of the full device stack. **b** HRTEM image. **c** Local magnification image showing the orthorhombic HZO polar phase (space group: $Pca2_1$). The interfacial layer IL at the TiN-HZO interface is structurally invisible. **c** corresponds to the marked region with pink dashed rectangular in **b**. Scale bars in **a**, **b** and **c** are 10 nm, 5 nm and 1 nm, respectively.

To accurately study the interface TiN-HZO from a chemical point of view, STEM-EELS measurements were performed using a probe-corrected microscope. In Figs. S6-S8, the chemical maps and profiles reveal that the IL between TiN and HZO corresponds to an oxynitride TiO_xN_y with an increasing oxygen content and decreasing N content towards HZO, and, eventually a TiO_2 layer with possibly some amount of Hf and Zr ($Ti_{1-x}Hf(Zr)_xO_2$). The thickness is about 2.0 nm. The IL is due to the prior oxidation of TiN while transferring the TiN layer from the sputtering deposition tool to the atomic layer deposition (ALD) one and, also, to the exposure of the surface to water in the first ALD cycles during HZO deposition. For comparison, the chemical analysis of the Al_2O_3 layer is shown in Fig. S8.

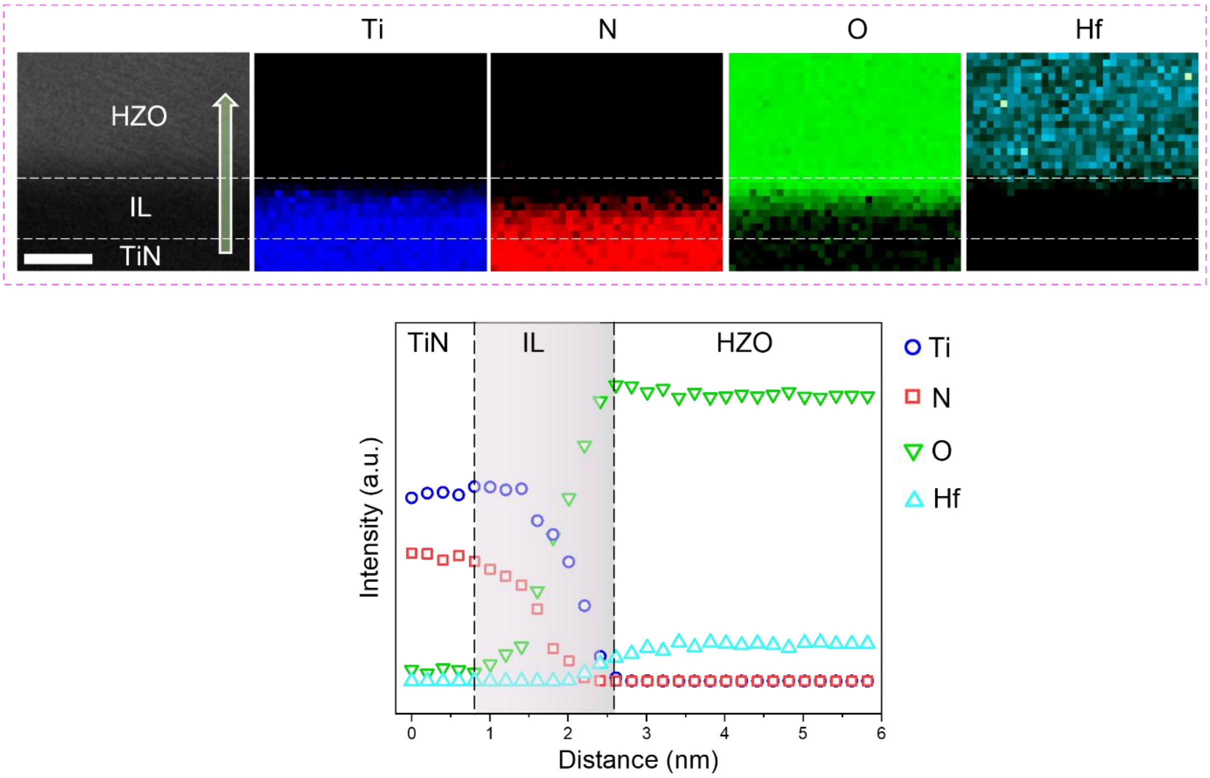

Fig. S6: EELS-STEM analysis showing the chemical composition maps of the TiN-HZO interface and the elemental profiles throughout the stack. IL, with a width of 1.8 ± 0.4 nm, represents the interfacial region, which is structurally indistinguishable. Scale bar is 2 nm.

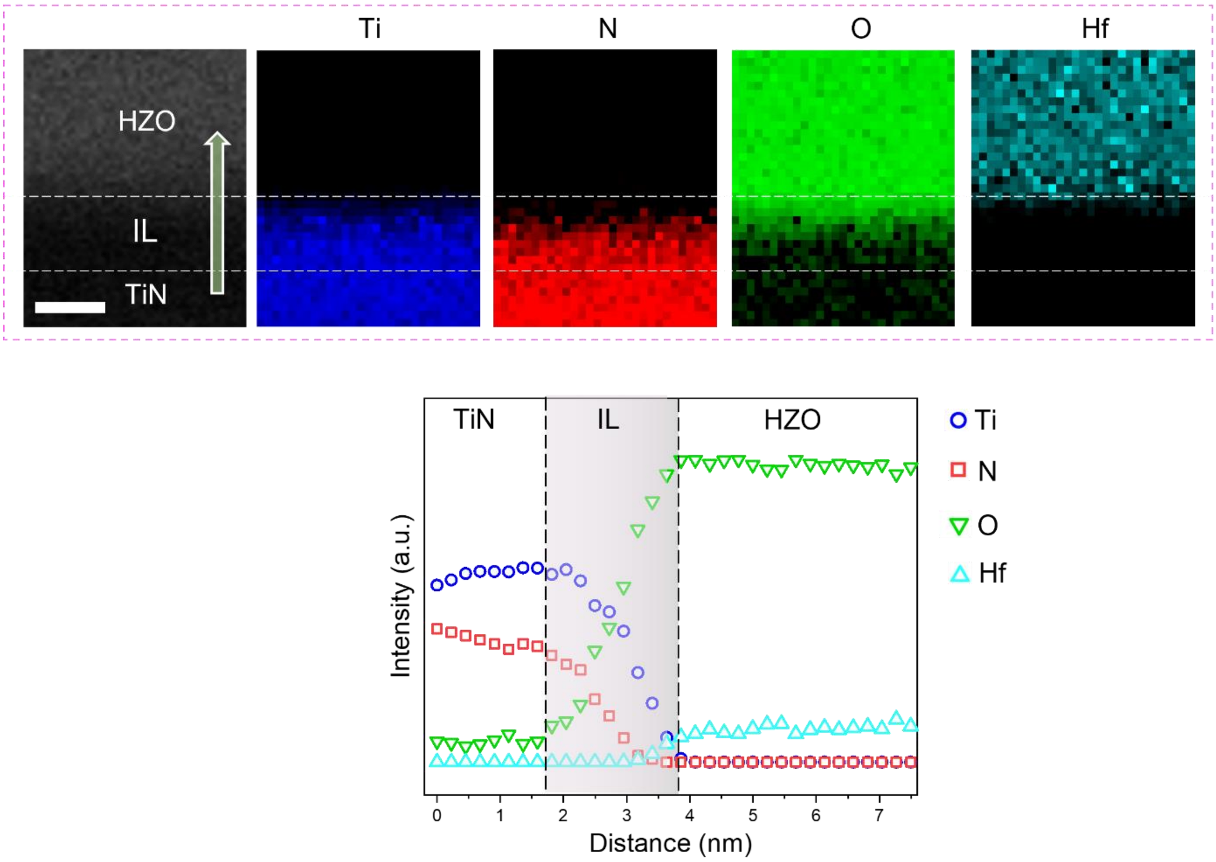

Fig. S7: EELS-STEM analysis showing the chemical composition maps of the TiN-HZO interface and the elemental profiles throughout the stack. IL, with a width of 2.1 ± 0.4 nm, represents the interfacial region, which is structurally indistinguishable. Scale bar is 2 nm.

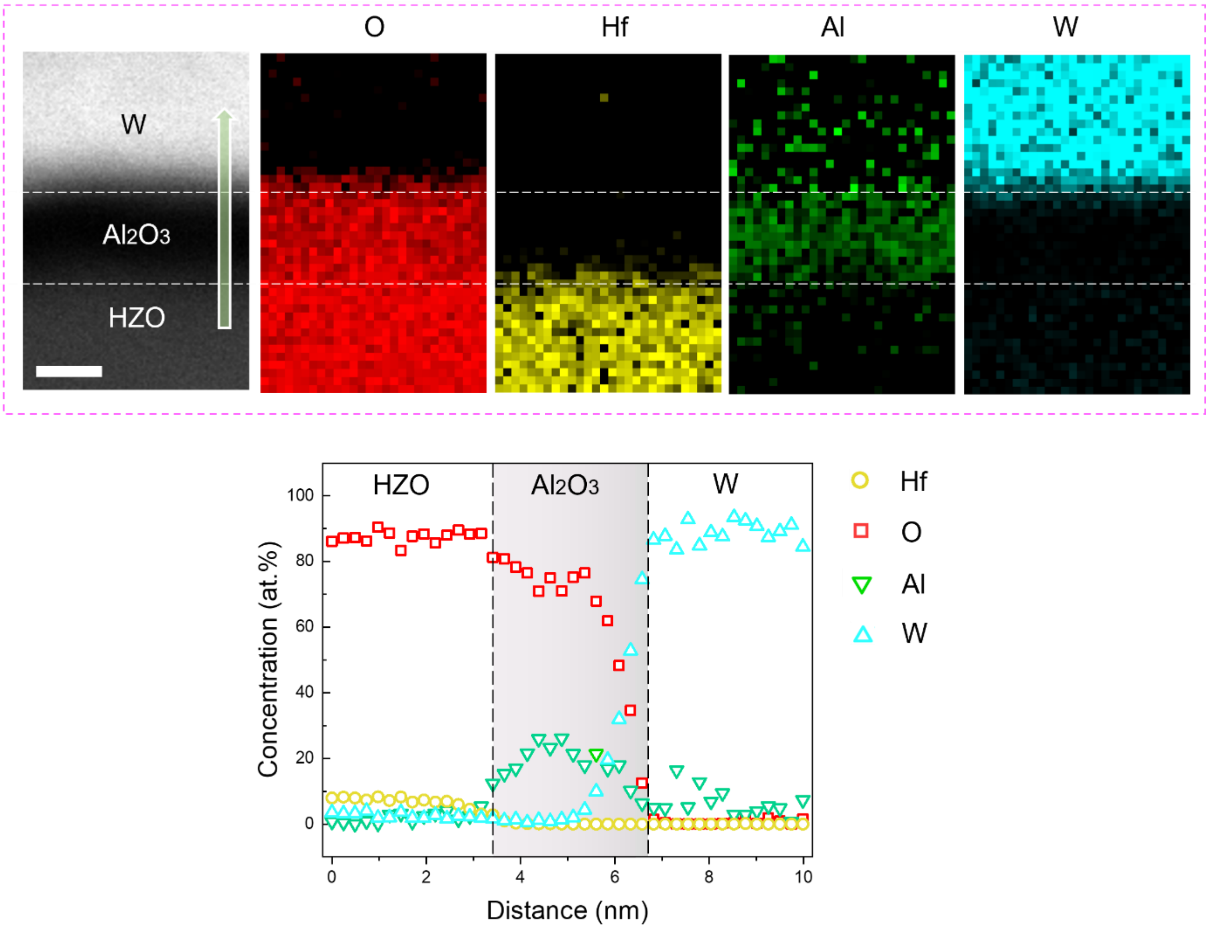

Fig. S8: EELS-STEM analysis showing the chemical composition maps and the elemental profiles throughout the HZO/Al_2O_3/W stack. The thickness of Al_2O_3 is measured to be 3.2 ± 0.4nm. Scale bar is 2 nm.

Figure S9 is adapted from Figs. 5b-d in the main article to help to better visualize the polarisation switching dynamics. The phase images of Fig. 5 have been averaged over 10 nm along the interfaces with a sliding averaging window.

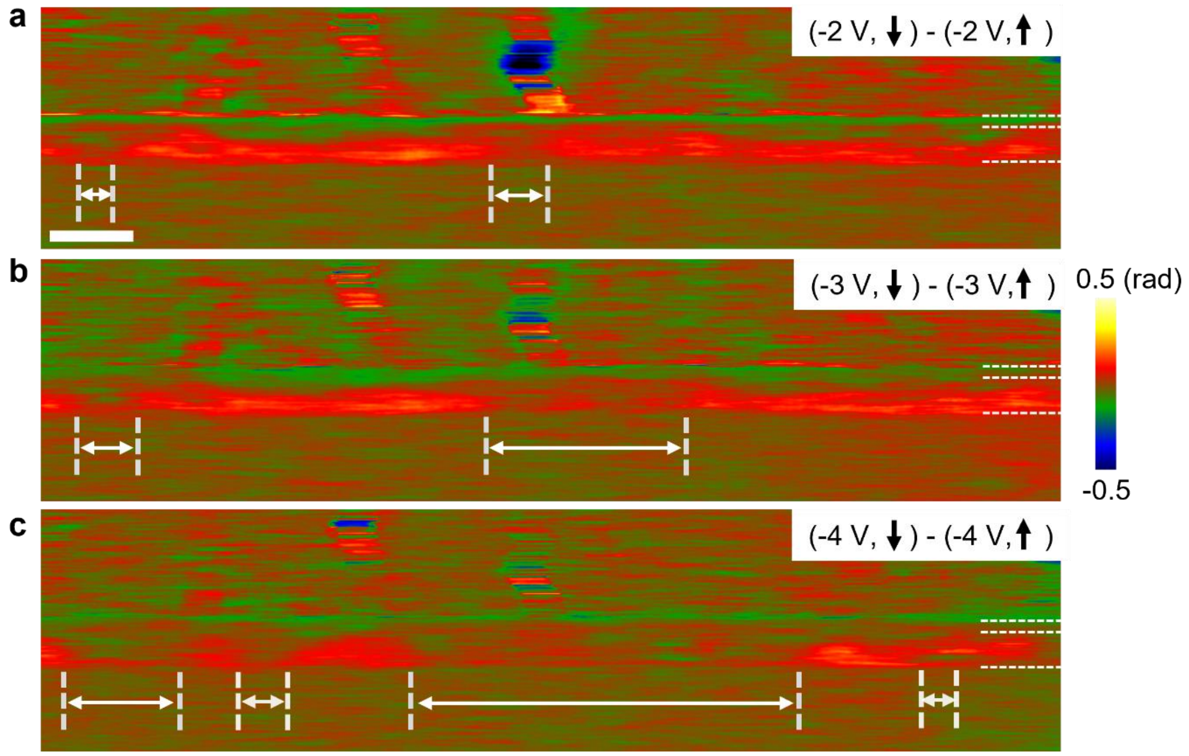

Fig. S9: Phase images highlighting the polarisation switching dynamics. a Difference of phase images under a bias of -2 V after switching the polarisation of HZO down (hologram E) then up (hologram J). **b** Difference of phase images (holograms F and I) at -3 V. **c** Difference of phase images (holograms G and H) at -4 V. All phase images were recorded in the same area and regions with white bidirectional arrows show the flipping of polarisation states. Scale bar is 20 nm.

Figure S10 shows some HRTEM images. We noticed that the lateral grain size is between 9 nm and 30 nm.

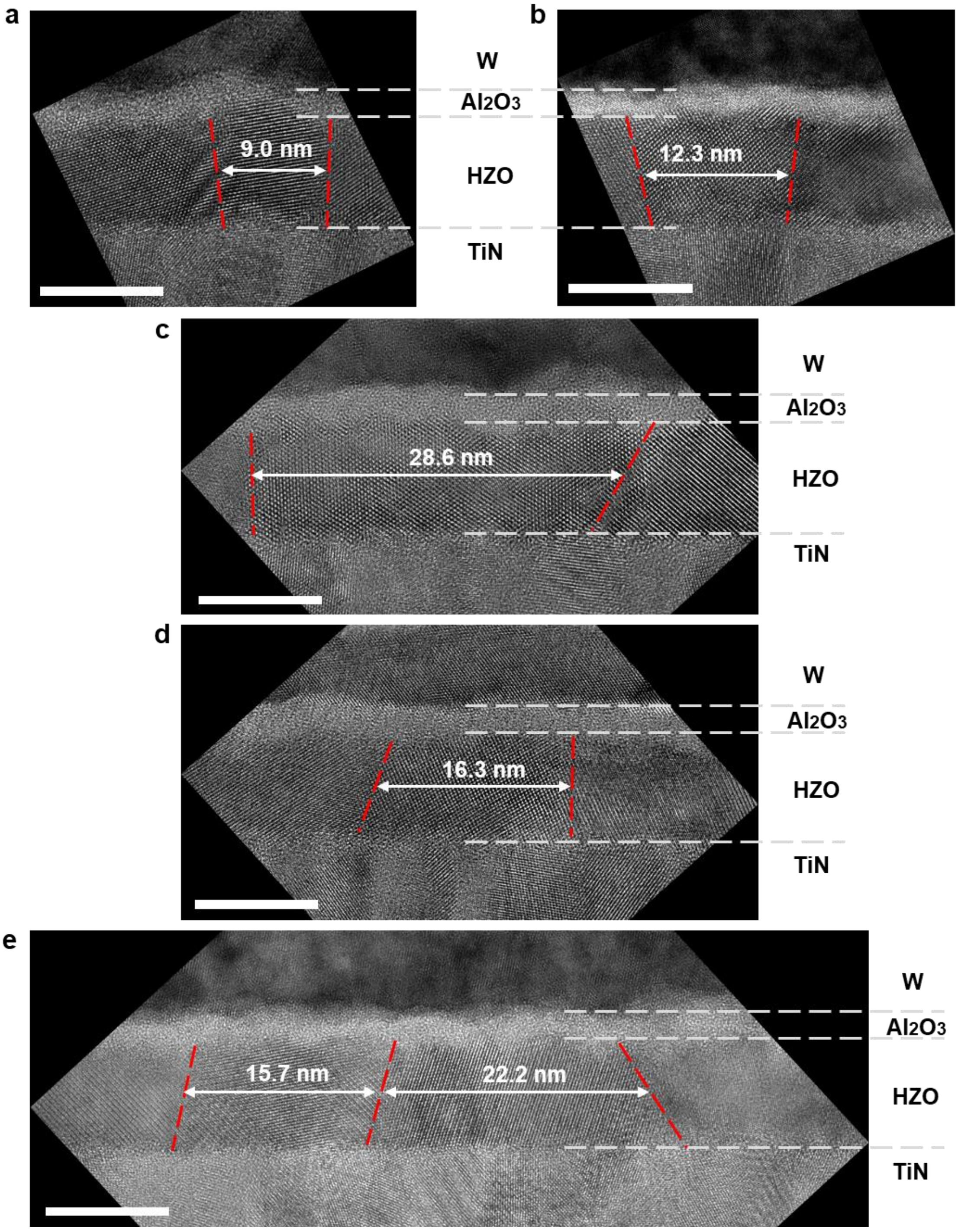

Fig. S10: HRTEM images showing the grains inside HZO layer. The lateral grain sizes are given in the images and the approximate grain boundaries are marked in red dashed lines. Scale bars are 10 nm.

Figure S11 presents the electric field measured at different locations of the phase images in Figs. 3a-b. The phase gradient was determined by linear regression in adjacent regions within the HZO layer, 14 nm (100 pixels) along the layers by 7 nm in the growth direction, corresponding to Figs. 3c-d. The phase gradient was then converted to the value of the electric field and corrected for the stray field. The error bar for an individual measurement is given by the standard deviation of the results, or 0.04 $MV \cdot cm^{-1}$.

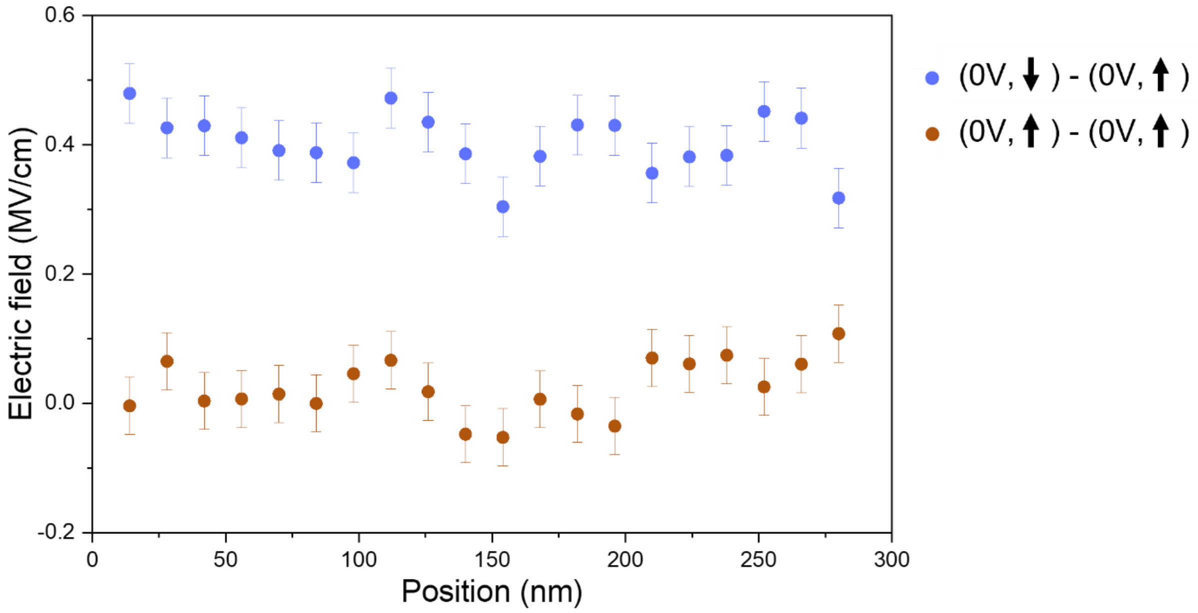

Fig. S11: The measured electric field and the error bars throughout the studied region (about 300 nm).

Figures S12 and S13 present the amplitude and phase images whilst the device is biased and when both electrodes are grounded (0 V). The phase images in Fig. S12 contain the MIP contribution. Figure S13 highlights that the amplitude images remain identical between the biased and unbiased states, unlike the phase images.

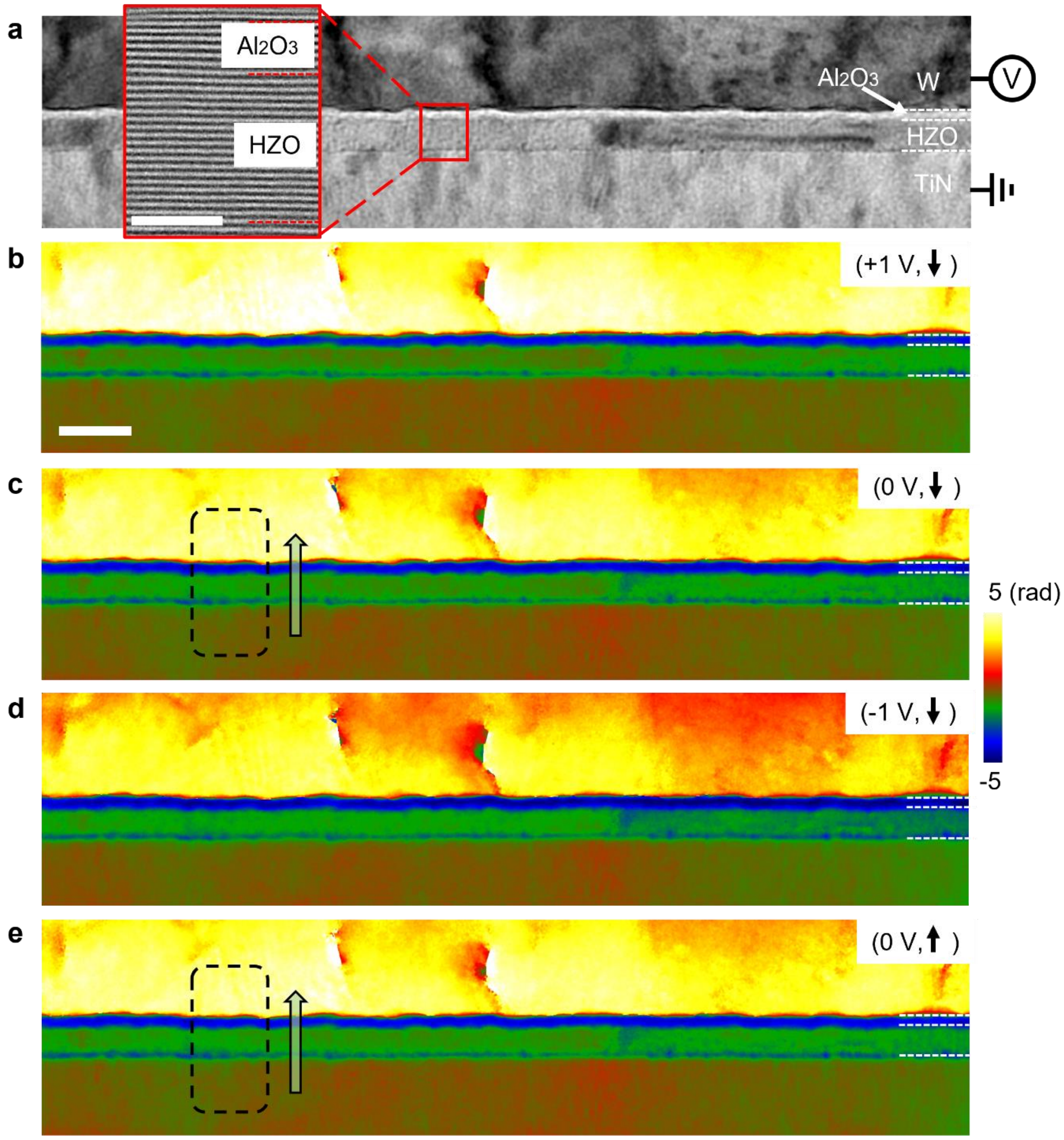

Fig. S12: Amplitude and phase images whilst the device is biased and when both electrodes are grounded (0 V). a Amplitude image showing the device architecture. The insert shows the holographic fringes corresponding to the area marked by the red rectangle. **b-e** Phase images obtained on the same region as **a** without removing the phase contribution recorded at 0 V. **b**, **c** and **d** have been acquired under a bias of +1 V, 0 V and -1 V, respectively, after switching down the polarisation of HZO (sweep from 0 to +5 V), while **e** was recorded at 0 V after switching up the polarisation (sweep from 0 to +5 V). Scale bar is 20 nm (5 nm for the insert).

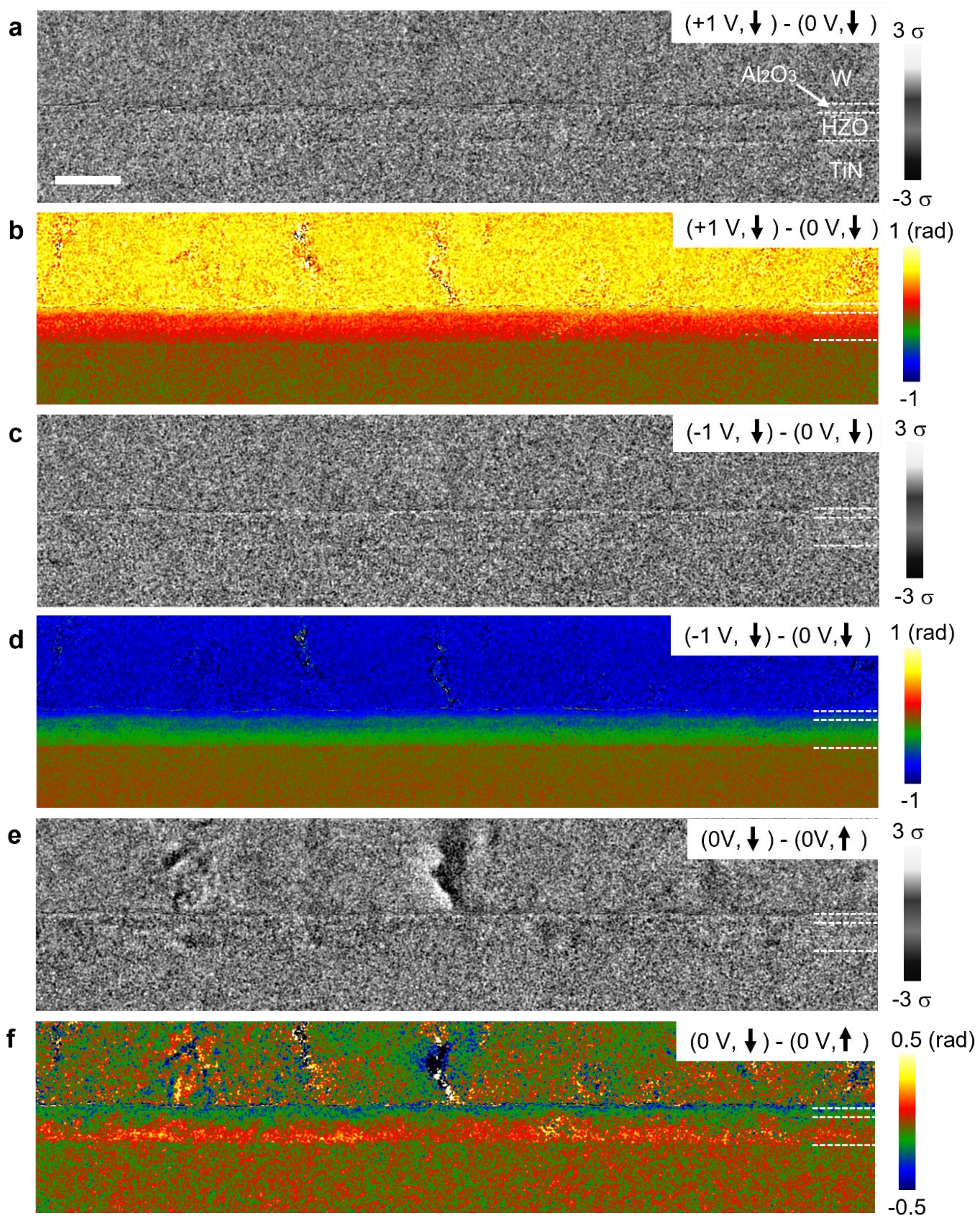

Fig. S13: Difference of amplitude and phase images acquired under different biases or after switching the polarisation of HZO down to up. All images correspond to the same area. **a** and **b** difference of amplitude and phase images, respectively, between +1 V (hologram B in Fig. 1c and phase image in Fig. S12b) and 0 V (hologram C and Fig. S12c) for two down states of the polarisation. **c** and **d** difference of amplitude and phase images, respectively, between -1 V (hologram D and Fig. S12d) and 0 V (hologram C and Fig. S12c) for two down states of the polarisation. **e** and **f** difference of amplitude and phase images, respectively, under a bias of 0 V after that the polarisation of HZO was switched down (hologram C and Fig. S12c) then up

(hologram L and Fig. S12e). σ on the gray scales used for the contrast corresponds to the root mean square value of the amplitude images. Scale bar is 20 nm.

Figure S14 presents two phase profiles extracted from Figs. S12c and S12e corresponding to grounded state with opposite polarisation of HZO. These profiles contain the MIP contribution and highlights the high sensitivity required to measure the variation of residual field.

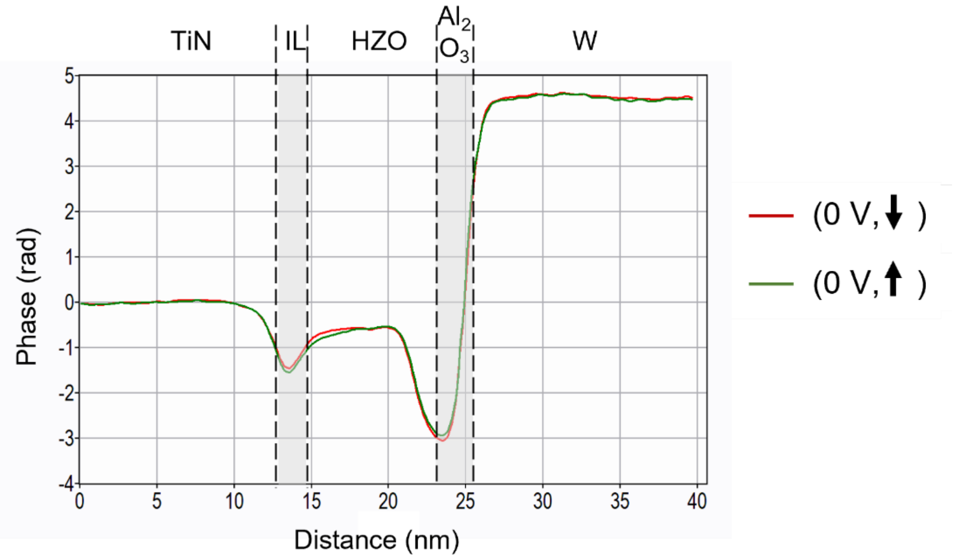

Fig. S14: Line profiles of the marked region in Figs. S12c and S12e recorded at 0 V after switching down the polarisation of HZO (sweep from 0 to +5 V) and switching up the polarisation (sweep from 0 to -5 V). Line profiles were obtained by averaging the phase over 20 nm parallel to the interface. We can note the strong MIP contribution compared to the residual electric field due to the insufficient number of charges to screen the polarisation bound charges.

Figures S15 to S17 illustrate the accuracy of the image realignment and data processing. All holograms used in this study were recorded on the exact same area. For removing MIP contribution and other static artefacts, phase images extracted from the holograms were subtracted each other. Figure S15 presents the amplitude and phase images for the entire study area (0 V after applying – 5 V). The *x* and *y* axes are those of the image. Figures S16 and S17 show the effect of misaligning a pixel in the 4 directions (+*x*, -*x*, +*y* and -*y*) parallel to the image axes for the amplitude and phase images respectively, when subtracting two grounded states with opposite polarisation. All images of one type (amplitude or phase) are displayed with the same contrast. σ on the gray scale in Fig. S16 corresponds to the root mean square value of the amplitude images, while the contrast set for the phase images in Fig. S16 is the same than the one used for Fig. 3 and Fig. 5 in the article.

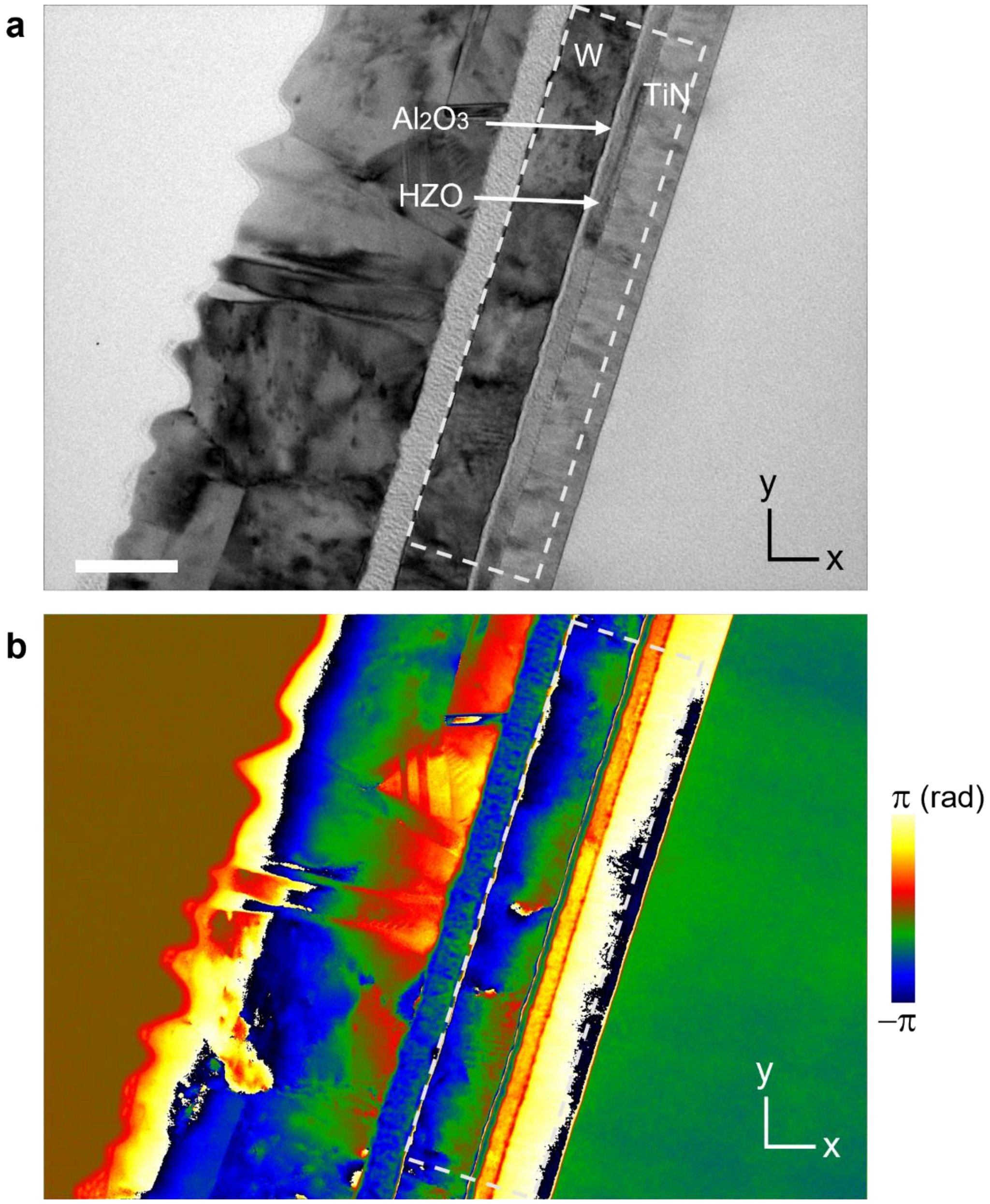

Fig. S15: Amplitude and phase images for the entire study area (0 V after applying – 5 V). **a** Raw amplitude image. **b** Phase images calculated from the complete hologram recorded with grounded electrodes (phase reference in the vacuum). Dotted rectangle indicates area extracted for the figures appearing in the paper. Axis indicate the axis of the raw images. Scale bar is 50 nm.

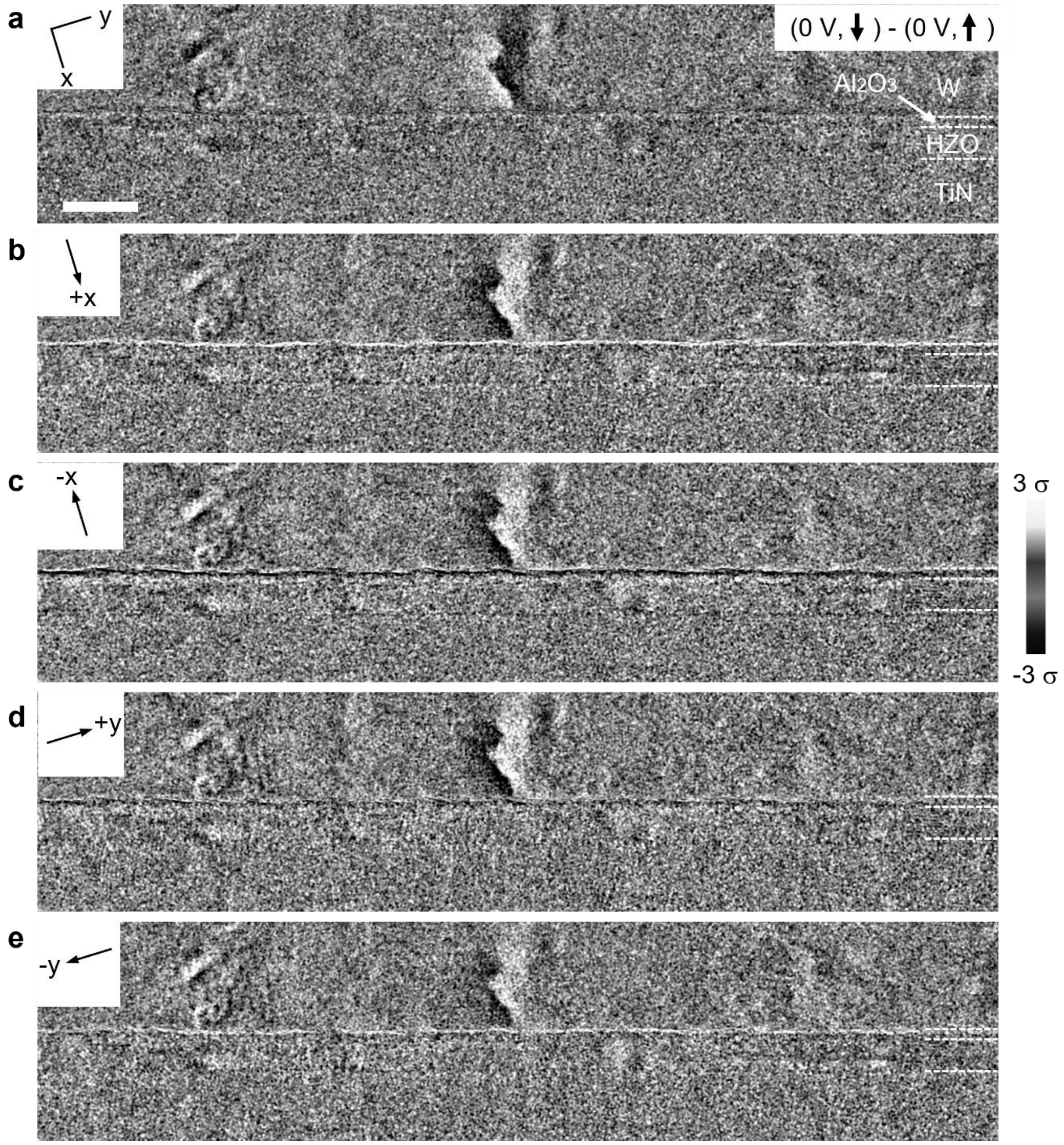

Fig. S16: Effect of misalignment of amplitude images when calculating their difference. Example taken from amplitude images obtained on the same area under a bias of 0 V after switching the polarisation of HZO down (hologram C and Fig. S12c) then up (hologram L and Fig. S12e). **a** Optimal alignment. **b-e** Displacement of one pixel between the images in the +x, -x, +y and -y directions, respectively, along the axis of the raw phase image shown in Fig. S15a. σ (on the gray scale used for the contrast) corresponds to the root-mean-square value of the amplitude images. Scale bar is 20 nm.

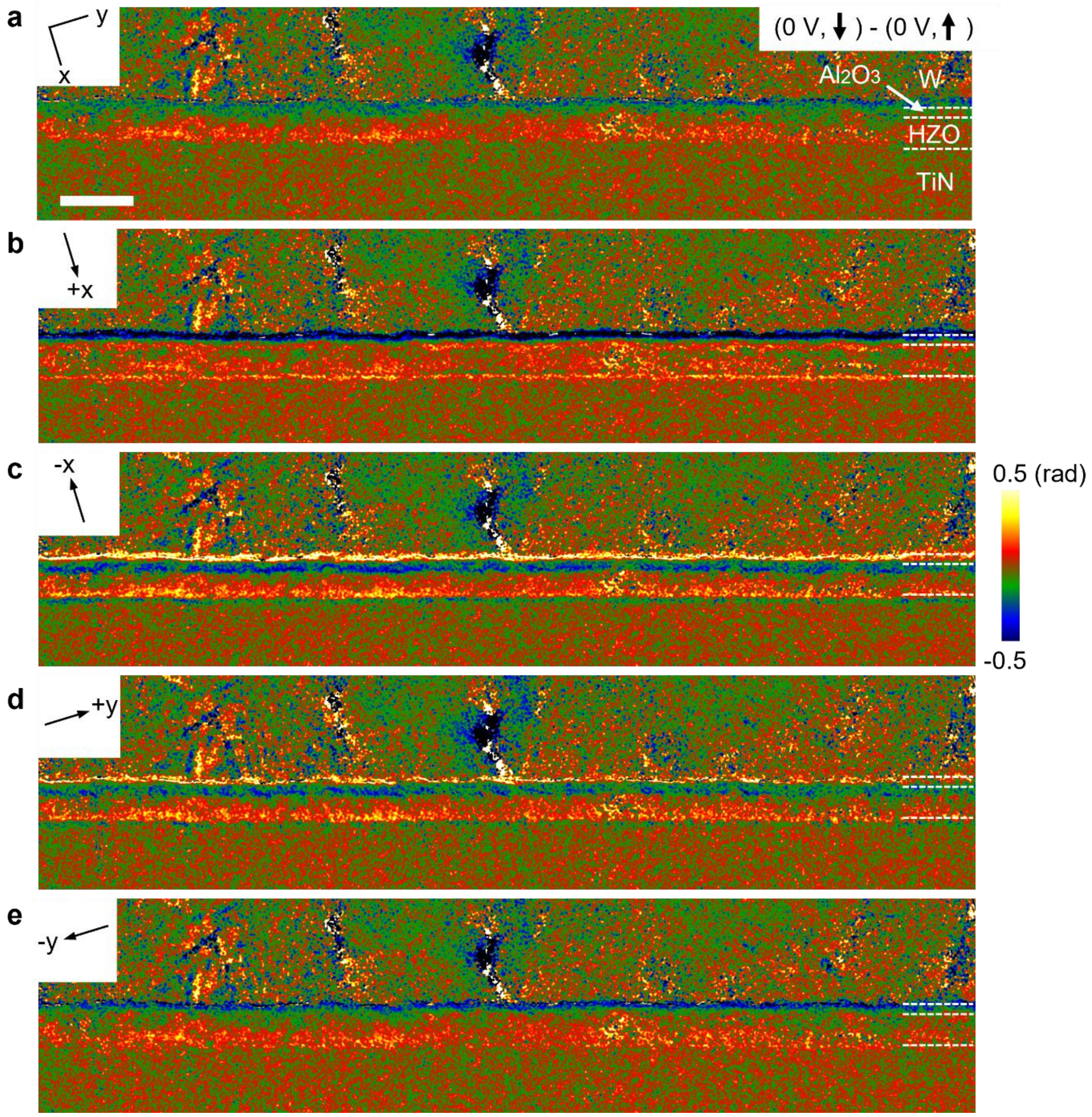

Fig. S17: Effect of misalignment due to drift when calculating the phase differences. Example taken from phase images obtained on the same area under a bias of 0 V after that the polarisation of HZO was switched down (hologram C and Fig. S12c) then up (hologram L and Fig. S12e). **a** Optimal alignment. **b-e** Displacement of one pixel between the images in the +x, -x, +y and -y directions, respectively, along the axis of the raw phase image shown in Fig. S15b. Scale bar is 20 nm.

Finite element modelling (FEM) simulations were conducted in a 2D geometry using COMSOL Multiphysics 5.6, a software specifically designed for studying the physical properties of systems, particularly those involving coupled physics problems. The stationary solver was applied to address the electrical distribution inside and outside the sample.

In Fig. S18a, the modelled region was square, 3.5 μm in both the propagation direction of the fast electron and the direction perpendicular to the interfaces. Not only the specimen geometry (for example, the lamella thickness, the length of each layer, the width of top and bottom electrodes) and the relative permittivity values, but also the charging layers at interfaces are incorporated into the models. The model geometry was determined using the amplitude image reconstructed from the electron hologram.

The electrostatic potential was calculated and integrated in the propagation direction to simulate the phase change of the fast electron according to the Eq. (1) in the main text. The simulated electrical potential map with a bias at 0 V was reported in Fig. S18b. Notice, in particular, how the stray field is limited to a region close to the sample surface.

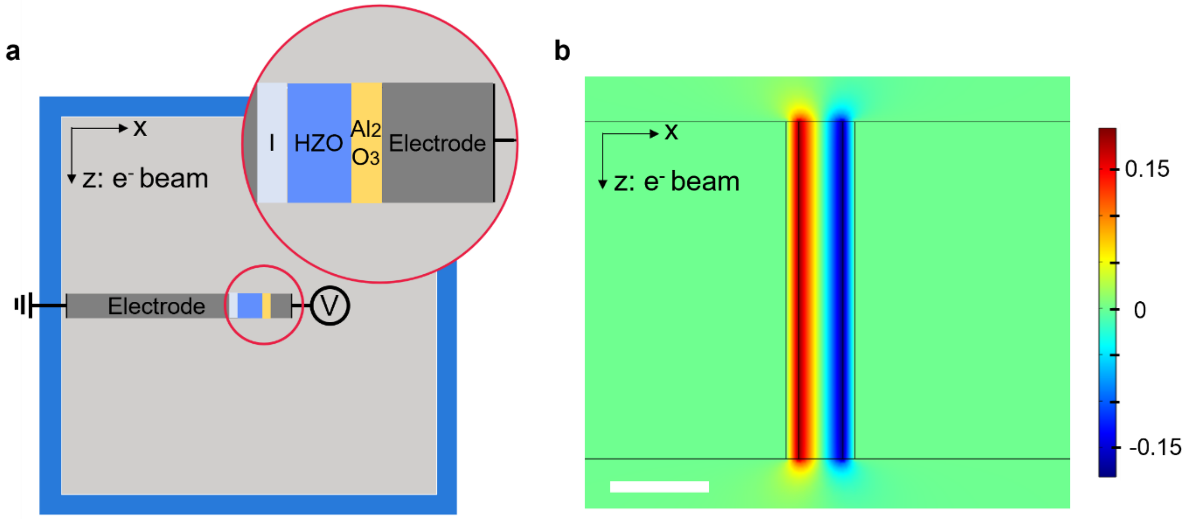

Fig. S18: Model used for the FEM calculation. a Illustration map showing the 2D structure. **b** the simulated electric potential with a bias at 0 V. The blue box in **a** describes the applied region for the simulation. The local magnification image, marked by a red circle, shows the active area of the device. Scale bar is 20 nm.

The measurement of the lamella thickness crossed by the electron beam is an essential part to quantify the residual field and the remaining charges. The quantification of the thickness value is performed following two steps. The initial estimation was obtained from electron holography by analyzing the phase due to the mean inner potential (MIP) in the silicon substrate (where the thickness is uniform after the FIB preparation) with respect to the vacuum (see Fig. S15b). In the absence of magnetic fields, the phase shift ϕ is proportional to the electrostatic potential V, encountered by the fast electrons along their trajectory:

$$\phi(x, y) = C_E \int V(x, y, z) dz \tag{1}$$

where x, y are the directions in the image plane, z the direction parallel to the electron beam and C_E the constant depending on the accelerating voltage of the microscope. $V(x, y, z)$ is a sum of MIP and the applied bias. If the composition is uniform along the electron beam direction (uniform MIP), and if no bias is applied, then Eq. (1) reduces further to:

$$\phi(x, y) = C_E V_{\mathrm{MIP}} t \tag{2}$$

where t is the sample thickness. Here, C_E takes a value of 6.53×10^6 rad/V/m at an accelerating voltage of 300kV [1]. The MIP value for Si crystals has been calculated to 11.47 V using Radi's binding model [2], 12.2 V for the Radi's nonbinding model [2] and 14.02 V with the D-T's nonbinding model [3]. From these values, we calculated an average value equal to 12.56 V. The mean phase shift in the Si substrate with respect to the vacuum has been measured to 5.43 rad. From the Eq. (2), we obtained an estimated thickness equal to 66 nm.

The second step involves the COMSOL simulation. We used this value as a starting point and we tried to find the best fitting with the experimental profiles at 1V and -1V. We identified that a thickness of 69 nm fits best with the experimental ones, and the errors was determined to be 3 nm considering the phase fluctuations in the electrode. The comparison between experimental profiles and simulated profiles are shown in Fig. S19. We finally used a sample thickness of 69 ± 3 nm.

References :

[1] R. E. Dunin-Borkowski, et al, Chapter 18: Electron holography. Science of Microscopy, **2007**, 1141-1195.

[2] D. Rez, P. Rez, I. Grant. Dirac–Fock calculations of X-ray scattering factors and contributions to the mean inner potential for electron scattering. *Acta Crystallographica Section A: Foundations of Crystallography*, **1994**, *50*(4), 481-497.

[3] P. T. Doyle, P. S. Turner. Relativistic Hartree–Fock X-ray and electron scattering factors. *Acta Crystallographica Section A: Crystal Physics, Diffraction, Theoretical and General Crystallography*, **1968**, *24*(3), 390-397.

Figure S19 presents a comparison between experimental simulated profiles with different thickness values for an applied bias of 1 V and -1 V. This fitting process is used to determine the thickness value of the lamella and the related error.

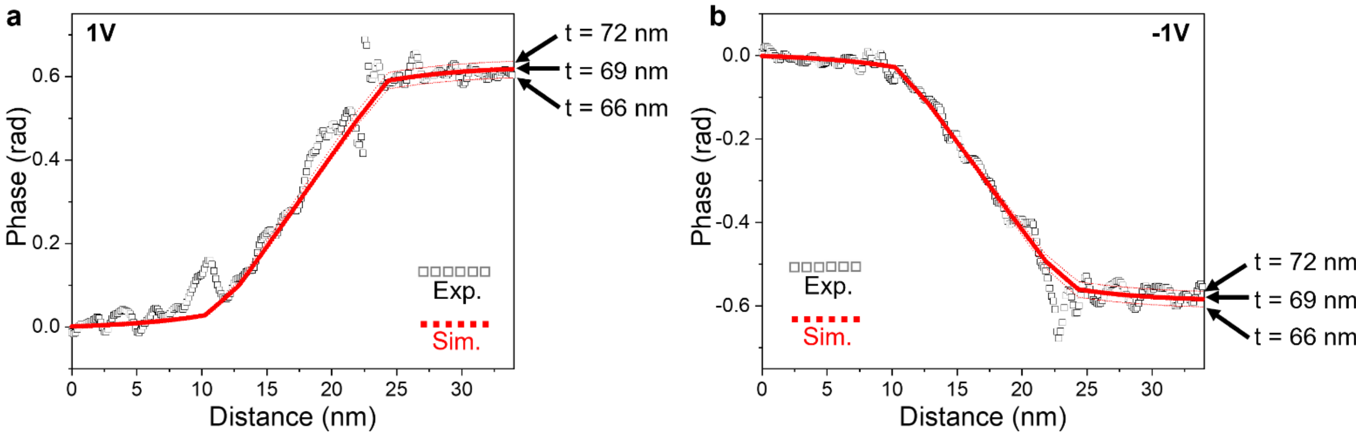

Fig. S19: Experimental and simulated profiles with different thickness values for an applied bias. a 1 V. **b** -1 V. The experimental profiles have been extracted in the same marked area in Fig. 3a on phase images after subtracting the phase image with grounded electrodes with the same polarisation state of HZO. Besides that, with a thickness of 69 nm, the two simulated profiles with thickness values of 66 nm and 72 nm are also shown.

In Fig. S20, we show the simulated phase profile (red), as well as the contributed ones from the stray field (blue) and internal field (green) at a bias of 0 V. The phase profiles were converted to electric potential by dividing by $C_E t$, where t is the thickness of the thin lamella (69 nm). This relation would correspond exactly to Equation (1) if all the field was within the sample. As we can see, this is almost exactly the case, as only 5% of the signal comes from the stray field outside the sample. Indeed, we can correct for the stray field to a very good approximation simply by reducing the measured phase and electric potential profiles by this amount (cf Methods). The residual error then comes from the uncertainty in the lamella thickness.

Simulations also show that the potential within the thin lamella is almost identical to the field that would be in an infinitely thick lamella. This was verified by comparing the potential within the thin lamella calculated above (green curve on Fig. S20) and the potential within a simulation of an infinitely thick lamella. The reason is that the total thickness of the layers (14 nm), where the electric field is concentrated, is much smaller than the thickness of the foil (69 nm). In such cases, the stray field is very small. Furthermore, the measurements were carried out at 0 V applied bias so there is no potential difference between the two electrodes. This explains the contrast with our previously published work [Physical Review Letters **139**, 137701 (2022)] where the distance between the two electrodes was comparable to the foil thickness and the measurements were carried out under applied bias.

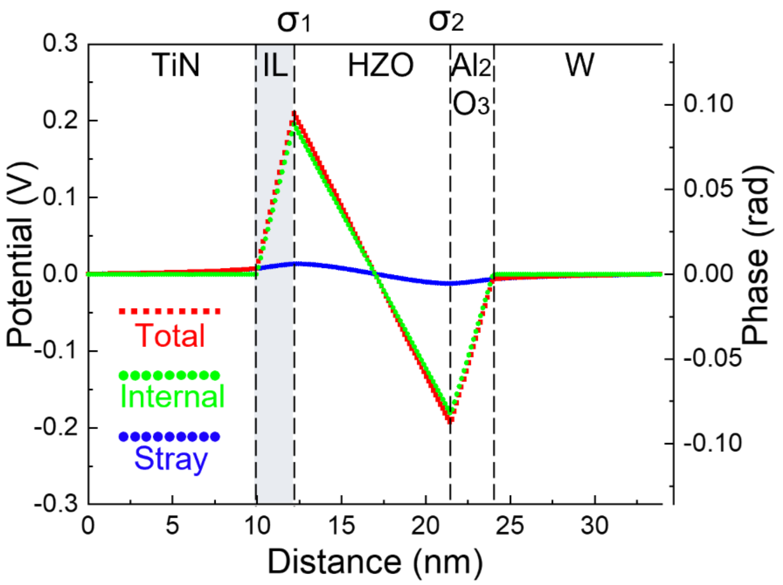

Fig. S20: Finite element method (FEM) modeling simulation of electric potential at a biasing of 0 V. The phase and potential profiles are shown for the total integrated field (red dashed), internal field (green dotted) and stray field (blue dotted). See text for conversion from phase profile to electric potential.
